# Magnetic Resonance Imaging Cooling-Reheating Protocol Indicates Decreased Fat Fraction via Lipid Consumption in Suspected Brown Adipose Tissue

**DOI:** 10.1371/journal.pone.0126705

**Published:** 2015-04-30

**Authors:** Elin Lundström, Robin Strand, Lars Johansson, Peter Bergsten, Håkan Ahlström, Joel Kullberg

**Affiliations:** 1 Department of Radiology, Uppsala University, Uppsala, Sweden; 2 Department of Information Technology, Uppsala University, Uppsala, Sweden; 3 Department of Medical Cell Biology, Uppsala University, Uppsala, Sweden; St. Joseph's Hospital and Medical Center, UNITED STATES

## Abstract

**Objectives:**

To evaluate whether a water-fat magnetic resonance imaging (MRI) cooling-reheating protocol could be used to detect changes in lipid content and perfusion in the main human brown adipose tissue (BAT) depot after a three-hour long mild cold exposure.

**Materials and Methods:**

Nine volunteers were investigated with chemical-shift-encoded water-fat MRI at baseline, after a three-hour long cold exposure and after subsequent short reheating. Changes in fat fraction (FF) and R_2_*, related to ambient temperature, were quantified within cervical-supraclavicular adipose tissue (considered as suspected BAT, denoted sBAT) after semi-automatic segmentation. In addition, FF and R_2_* were quantified fully automatically in subcutaneous adipose tissue (not considered as suspected BAT, denoted SAT) for comparison. By assuming different time scales for the regulation of lipid turnover and perfusion in BAT, the changes were determined as resulting from either altered absolute fat content (lipid-related) or altered absolute water content (perfusion-related).

**Results:**

sBAT-FF decreased after cold exposure (mean change in percentage points = -1.94 pp, P = 0.021) whereas no change was observed in SAT-FF (mean = 0.23 pp, P = 0.314). sBAT-R_2_* tended to increase (mean = 0.65 s^-1^, P = 0.051) and SAT-R_2_* increased (mean = 0.40 s^-1^, P = 0.038) after cold exposure. sBAT-FF remained decreased after reheating (mean = -1.92 pp, P = 0.008, compared to baseline) whereas SAT-FF decreased (mean = -0.79 pp, P = 0.008, compared to after cold exposure).

**Conclusions:**

The sustained low sBAT-FF after reheating suggests lipid consumption, rather than altered perfusion, as the main cause to the decreased sBAT-FF. The results obtained demonstrate the use of the cooling-reheating protocol for detecting changes in the cervical-supraclavicular fat depot, being the main human brown adipose tissue depot, in terms of lipid content and perfusion.

## Introduction

There are two main types of mammal adipose tissue: white adipose tissue (WAT) and brown adipose tissue (BAT), formed by white and brown adipocytes, respectively. Whereas WAT primarily serves as lipid storage, BAT generates heat by non-shivering thermogenesis. The use of BAT activation as a defense against hypothermia has long been considered important in small mammals but has in humans been regarded as significant mainly in newborns. However, it is now well established that BAT is prevalent also in young adults (in the twenties and thirties) [1], with the main depot being the cervical-supraclavicular, and can be activated to contribute to non-shivering thermogenesis during cold exposure [2]. Despite its modest amount in humans, BAT has a therapeutic potential for obesity and associated diseases (e.g. type 2-diabetes) and has therefore gained research interest [3, 4].

Positron emission tomography combined with computed tomography (PET/CT) is the predominant technique for BAT imaging in humans [5–8]. Although being essentially non-invasive and providing high sensitivity in detecting active BAT, research with PET/CT is restricted due to exposure to ionizing radiation. In this context, magnetic resonance imaging (MRI) has been proposed as a non-ionizing imaging alternative and complement to PET/CT [9–16]. As the cervical BAT depot in human adults has been observed to contain a mixture of brown adipocytes and white adipocytes [17], BAT imaging is expected to be challenged by partial volume effects.

Water-fat MRI, based on chemical shift imaging [18], is one of the promising MRI techniques for studying human BAT [9–16, 19]. Multi-echo acquisition enables quantitative and simultaneous estimations of fat fraction (FF) and transversal signal decay R_2_*, where *FF = F/(F+W)* is calculated from fat signal (F) and water signal (W) and where *R*_*2*_ * = 1/T_*2*_ * is related to the T_2_* relaxation time [20]. As reported from studies in children and young adults [9–12, 14, 19], BAT might be distinguished from WAT by assuming a comparably low BAT-FF due to a lower intra-cellular lipid content and a denser capillary network [21]. As previously studied in children and adolescents [10, 11], BAT might also be separated from WAT by differences in R_2_* that probably reflect the higher iron content in BAT due to the numerous capillaries and mitochondria [21]. Since cold-induced BAT activity likely is associated with lipid consumption [2, 22] and increased perfusion [22–24] (possibly along with a perfusion-related increase in blood volume) a decrease in BAT fat content relative to water content (i.e. BAT-FF) might be observed after cold exposure. In addition, cold-activated BAT is discussed to be associated with altered blood oxygenation [11, 12] along with changes in perfusion and blood volume [12] that could be reflected by changes in R_2_*. Previous PET/CT-studies report higher CT Hounsfield units (HU) of active BAT as compared to inactive BAT or WAT [22, 25]. Additionally, one study reports intra-subject increase in CT HU with BAT activation during a three-hour long cooling protocol [2]. These results suggest a cold-induced decrease in BAT fat content relative to water content that could be detected and quantified using water-fat MRI. Although a recent MRI study in newborns shows FF differences in BAT regions between hypothermia treated patients and controls [19], comparisons of intra-subject FF data acquired before and after cold exposure have not been shown. In addition, while lipid consumption is suggested as the primary cause to the higher CT HUs observed in BAT under activated conditions as compared to under non-activated conditions [2, 22], increased perfusion (and blood volume) could also contribute to this difference [22]. The possible effect of perfusion, on the decreased BAT fat content during activity, has not yet been assessed. Although BAT response time, e.g. regarding perfusion alterations upon activation-inactivation, is not known it is probably relatively rapid (at the scale of minutes). In a recent study, using dynamic T_2_*-weighted imaging of human BAT during 5–15 min recurrent cooling-heating intervals, changes in T_2_*-weighted signal are observed to coincide temporally with the cold exposure [12]. The origin of these signal fluctuations is not fully understood but it is probably associated with changes in tissue perfusion and blood oxygenation occurring during BAT activation-inactivation. To distinguish between the effects of lipid content and perfusion, two assumptions were made in the present study. Firstly, perfusion was assumed to be rapidly regulated and therefore likely to regress by short reheating (approximately 15 min) after cold exposure. Secondly, lipid content was assumed to be slowly regulated and not regressing during the same short reheating.

The purpose of the present study was to evaluate whether a cooling-reheating protocol, coupled with FF and R_2_* MRI measurements, could be used to detect changes in lipid content and perfusion in cervical-supraclavicular adipose tissue (suspected BAT, denoted sBAT) after three hours of mild cold exposure. A reduced sBAT-FF after cold exposure indicated cold-induced BAT activity. As the lowered sBAT-FF did not normalize after short reheating, lipid consumption was considered as a more likely cause than perfusion to this change in FF. The overall results demonstrate the use of the water-fat MRI cooling-reheating protocol for detecting changes in cervical-supraclavicular adipose tissue lipid content and perfusion.

## Materials and Methods

### Subjects

Nine volunteers (five males, four females, age: 30±5 years, age range: 22–37 years, body mass index (BMI): 23.2±2.5 kg/m^2^, S1 Table) gave informed written consent to participate. The study was approved by the Regional Ethical Review Board in Uppsala.

### Study design

**Fig 1:**
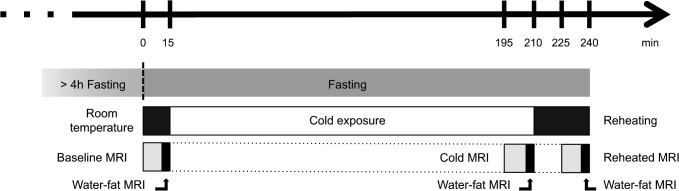
The *Cooling-reheating protocol* design.

The study protocol, denoted the *Cooling-reheating protocol* (Fig 1), consisted of three consecutive water-fat MRI scans performed under three different conditions: *Baseline MRI* (room-temperature) → *Cold MRI* (after three hours of cold exposure) → *Reheated MRI* (after short reheating). The *Baseline MRI* was used to study whether cervical-supraclavicular adipose tissue (suspected to contain BAT and thereby denoted sBAT) could be distinguished from WAT (adipose tissue not suspected to contain BAT), through the presumed differences in FF and R_2_*. Metabolically active BAT, within sBAT, was targeted by studying changes between the *Baseline MRI* and the *Cold MRI*. The *Reheated* MRI was proposed to be useful for studying how eventual changes in FF and R_2_* during cold exposure could be related to alterations in lipid content and perfusion.

In detail, the *Cooling-reheating protocol* consisted of the following steps: 1) After fasting for at least four hours, the subjects were dressed in standardized clothing (underwear, socks and patient smock). 2) Imaging of the subjects, according to a standardized experimental set-up and imaging protocol (including water-fat MRI), was performed (*Baseline MRI*). 3) The subjects were exposed to a three-hour long cold exposure while sitting on a chair inside a dedicated room of temperature 18.8±0.5°C, obtained using a portable air conditioning system. 4) Imaging was performed in the same manner as in the *Baseline MRI* but with addition of a bottle of cold water on the calves and a standardized increase in airflow through the magnet tunnel to maintain cooling (*Cold MRI*). 5) Reheating was performed directly following the *Cold MRI*, with the subjects still lying on the MR patient table (in front of the magnet) for approximately 15 min, using blankets and a bottle of warm water on the calves. 6) Reheating continued during subsequent imaging (*Reheated MRI*).

### Procedure study

Eight of the nine subjects (age: 29±5 years, age range: 22–37 years, BMI: 23.5±2.5 kg/m^2^) were reinvestigated for a *Procedure study* during a separate follow-up visit. This study was performed to determine whether the unexpected reduction in posterior subcutaneous WAT-FF, observed between the *Cold MRI* and the *Reheated MRI*, was related to ambient temperature or to the supine position maintained by the subjects between the two scans.

In the *Procedure study*, the *Cooling-reheating protocol* steps 4–6 were performed without prior cold exposure and without reheating. The following modifications were made with respect to the original protocol: 1) The subjects had fasted for a minimum of seven hours (to account for the four-hour fasting before cold exposure and the three-hour fasting during cold exposure). 2)-3) As no cold exposure was applied, these steps were omitted 4) Neither a bottle of cold water, nor an increased airflow was used during imaging. 5) During the resting period on the MR patient table, no heating accessories (bottle of water, blanket) were used 6) No heating accessories were used during imaging.

### Image acquisition

Image acquisition was performed on a clinical whole-body 1.5 T MR system (Philips Healthcare, Best, The Netherlands). Water-fat images were obtained using a 3D multi gradient echo sequence and a 16 channel neurovascular receive coil (Philips Healthcare, Best, The Netherlands) during a 4 min 40 s scan in free breathing. To reduce respiratory artifacts the subjects were instructed to breath shallowly. The following scan parameters were used: axial acquisition, repetition time/echo time 1/echo time spacing = 32.7/1.68/2.87 ms, 6 unipolar echoes, flip angle = 6°, water-fat shift = 0.25 voxels, SENSE acceleration = 1.5 in anterior-posterior (fold-over) direction, field of view (right-left × anterior-posterior × feet-head) = 480×200×50 mm^3^, acquired/reconstructed voxel size = 1.0×1.0×2.0 mm^3^, 25 slices, number of signal acquisitions = 2. The flip angle was chosen small to reduce T_1_-weighting. No contrast agents or pharmacological agents were administered. Imaging was focused on the bilateral cervical-supraclavicular fat depot and the imaging volume was positioned so that the top 3–4 slices typically were placed above the shoulders of the subjects.

### Image reconstruction

Coregistered water images, fat images, FF maps and R_2_* maps were obtained through a 3D reconstruction of acquired multi-echo data, using an in-house software. The water-fat separation algorithm consisted of a multi-scale version of a previously described method [20] and was based on a nine-peak fat resonance model, a single R_2_* estimation (decoupled determination) and a regularization parameter μ = 10.

### Image analysis

**Fig 2:**
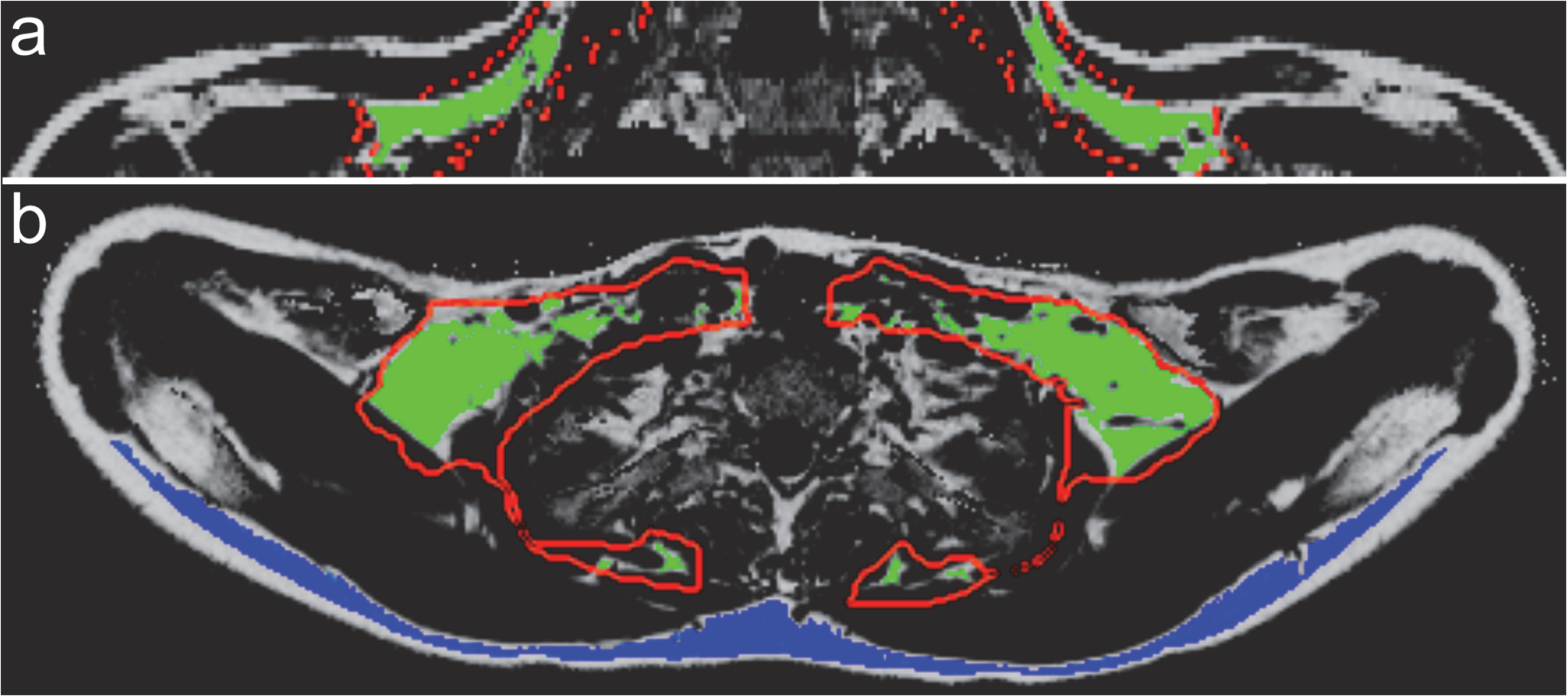
Segmentation of cervical-supraclavicular and subcutaneous adipose tissue. A coronal (a) and axial (b) fat fraction (FF) map showing the manually delineated crude cervical-supraclavicular (considered as suspected brown adipose tissue, denoted sBAT) volume of interest (VOI) with a red contour, the final segmented sBAT VOI in green and the final segmented posterior subcutaneous adipose tissue (SAT) VOI in blue. Segmentation of the sBAT and SAT VOIs was accomplished by exclusion criteria on FF, R_2_* and by erosion.

A crude segmentation of the sBAT volumes of interest (sBAT VOIs) was performed manually on the baseline FF maps. The sBAT VOIs were defined to include adipose tissue located between the clavicula and the scapula and to exclude subcutaneous adipose tissue, bone marrow, paravertebral fat and intramuscular fat (Fig 2). Transfer of the sBAT VOIs from the baseline FF maps to the remaining data sets was accomplished by image registration. Within the manually segmented volume, an automatic segmentation was applied to isolate tissue with a FF ≥40% (removal of non-fatty tissue) and a R_2_* ≤50 ms (reduction of partial volume effects caused by boundary voxels between adjacent tissues). 3D erosion with a six-neighborhood structuring element was also applied to reduce partial volume effects generated between adjacent tissues (supporting information in S1 Text).

Volumes of WAT were defined fully automatically in posterior subcutaneous adipose tissue (SAT VOIs) without using spatial registration. The same criteria regarding erosion and range limits on FF and R_2_*, as were applied to the sBAT VOIs, were applied to the SAT VOIs (supporting information in S1 Text).

To validate the image registration, performed for sBAT VOI transfer, results obtained with registered sBAT VOIs were compared to results obtained with manually defined sBAT VOIs. For each subject, manual delineation in the *Baseline*, *Cold* and *Reheated MRI* data sets was performed on a side-by-side manner to obtain as similar outlining as possible.

Statistical analysis was performed using Statistica 12 (StatSoft Scandinavia AB, Uppsala, Sweden) for Windows. Reported data were expressed as means ± standard deviations. Differences and changes between intra-subject measurements were compared using Wilcoxon matched pairs test. *P* values <0.05 were considered statistically significant.

## Results

**Fig 3:**
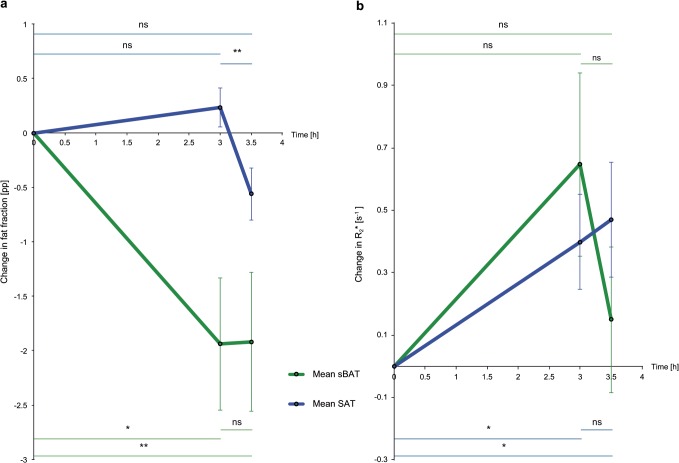
Changes in fat fraction and R_2_* of cervical-supraclavicular and subcutaneous adipose tissue during the *Cooling-reheating protocol*. Plots illustrating changes in (a) fat fraction (FF) and (b) R_2_* of cervical-supraclavicular adipose tissue (considered as suspected brown adipose tissue, denoted sBAT) and subcutaneous adipose tissue (SAT). The fat fraction changes are expressed in percentage points (pp). The time points correspond to the *Baseline MRI* (t = 0), the *Cold MRI* (t = 3h) and the *Reheated MRI* (t = 3.5h). Values plotted represent group means (of individual volume of interest (VOI) means) and standard error of the group means. *p<0.05; **p<0.01; (ns) non-significant.

**Fig 4:**
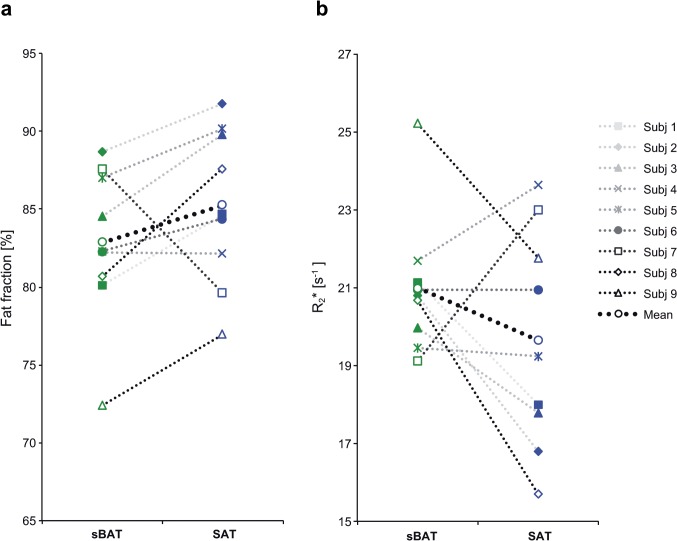
Differences in fat fraction and R_2_* between cervical-supraclavicular and subcutaneous adipose tissue at baseline. Measurements of (a) fat fraction (FF) and (b) R_2_* in cervical-supraclavicular adipose tissue (considered as suspected brown adipose tissue, denoted sBAT) and subcutaneous adipose tissue (SAT) in individual subjects from the *Baseline MRI*. Values plotted correspond to volume of interest means.

FF and R_2_* values in sBAT and SAT, during the *Cooling-reheating protocol*, are summarized in Table 1 (supporting information in S2 Table). Changes in FF and R_2_* during the protocol are graphically presented in Fig 3. FF and R_2_* values in sBAT and SAT from the *Baseline MRI* are presented in Fig 4. Changes in FF and R_2_* between the *Cold* and *Reheated MRI*, in addition to changes between the MRIs in the *Procedure study*, are summarized in Table 2 (supporting information in S2 and S3 Tables).

**Table 1:** Summary of fat fraction (FF) and R_2_* measurements.

Measurement	Baseline	Cold	Reheated
**sBAT-FF [%]**	82.8±5.0 (72.4–88.7)	80.9±6.1 (67.3–87.6)	80.9±6.5 (66.3–86.9)
**SAT-FF [%]**	85.2±5.1 (77.0–91.8)	85.5±4.8 (77.2–92.2)	84.7±4.7 (76.4–91.0)
**sBAT-R**_**2**_ * [s ^**-1**^ **]**	21.0±1.8 (19.1–25.2)	21.6±2.1 (19.3–26.3)	21.1±2.1 (19.4–25.9)
**SAT-R**_**2**_ * [s ^**-1**^ **]**	19.6±2.8 (15.7–23.6)	20.0±2.7 (16.4–23.7)	20.1±3.0 (16.6–25.0)

Note—Data on cervical-supraclavicular adipose tissue (considered as suspected brown adipose tissue, denoted sBAT) and subcutaneous adipose tissue (SAT) during the *Cooling-reheating protocol* (n = 9). Measurements reported as group means ± standard deviations (ranges) of individual volume of interest (VOI) means.

**Table 2:** Changes in fat fraction (FF) and R_2_ ^*^.

Measurement	Cold-Reheated	Procedure study	Difference
**∆sBAT-FF [pp]**	0.02 (ns)	-0.02 (ns)	(ns)
**∆SAT-FF [pp]**	-0.79 **	-0.75 *	(ns)
**∆sBAT-R**_**2**_ * [s ^**-1**^ **]**	-0.50 (ns)	0.25 (ns)	(ns)
**∆SAT-R**_**2**_ * [s ^**-1**^ **]**	0.07 (ns)	0.02 (ns)	(ns)

Note—Data on cervical-supraclavicular adipose tissue (considered as suspected brown adipose tissue, denoted sBAT) and subcutaneous adipose tissue (SAT) during the *Cooling-reheating protocol* (n = 9) and the *Procedure study* (n = 8). Result column 1–2: Measurements reported as group mean changes between the intra-protocol measurements with statistical significance of the changes given in parentheses. Fat fraction changes expressed in percentage points (pp). Result column 3: Statistical significance of differences between the changes obtained from the two protocols.

* p<0.05;

** p<0.01;

(ns) non-significant.

### Cooling-reheating protocol

sBAT-FF decreased by an average of -1.94±1.83 percentage points (pp) (P = 0.021) after the three-hour long cold exposure, whereas no change was observed in SAT-FF (0.23±0.53 pp, P = 0.314) (Table 1, Fig 3A). sBAT-R_2_* tended to increase (0.65±0.88 s^-1^, P = 0.051) and SAT-R_2_* increased by 0.40±0.46 s^-1^ after cold exposure (P = 0.038) (Table 1, Fig 3B). After reheating, sBAT-FF remained decreased (-1.92±1.92 pp, P = 0.008 compared to the *Baseline MRI* and 0.02±0.99 pp, P = 0.953 compared to the *Cold MRI*) (Table 1, Fig 3A). SAT-FF decreased in all individuals after reheating, by an average of -0.79±0.27 pp (P = 0.008 compared to the *Cold MRI*) (Table 1, Fig 3A). A weak trend of sBAT-R_2_* normalization was observed after reheating (0.15±0.70 s^-1^, P = 0.441 compared to the *Baseline MRI* and -0.50±1.33 s^-1^, P = 0.173 compared to the *Cold MRI*) (Table 1, Fig 3B) whereas SAT-R_2_* remained increased (0.47±0.55 s^-1^, P = 0.028 compared to the *Baseline MRI* and 0.07±0.49 s^-1^, P = 0.767 compared to the *Cold MRI*) (Table 1, Fig 3B).

At baseline (*Baseline MRI*), sBAT-FF tended to be lower than SAT-FF (-2.41±4.36 pp, P = 0.139). In addition, there was a weak trend of higher sBAT-R_2_* than SAT-R_2_* (1.35±2.96 s^-1^, P = 0.173). Two subjects (Subj 4 and 7, Fig 4) showed higher FF and lower R_2_* in sBAT than SAT, whereas the rest of the subjects showed lower FF and higher R_2_* in sBAT than SAT.

### Procedure study

There was no change observed in sBAT-FF between the two scans (-0.02±0.84 pp, P = 1.000) whereas SAT-FF decreased in all subjects, by an average of -0.75±0.64 pp (P = 0.012) (Table 2). No change was observed between the two scans in neither sBAT-R_2_* (0.25±0.91 s^-1^, P = 0.327) nor SAT-R_2_* (0.02±0.37 s^-1^, P = 0.779) (Table 2). Neither was there any difference observed in FF and R_2_* changes between *Cold MRI—Reheated MRI* and the two MRIs in the *Procedure study* (Table 2).

### Validation of registration

No statistically significant differences were observed between sBAT-FF results obtained from registered sBAT VOIs and from manually outlined sBAT VOIs (S2 and S4 Tables). Regarding sBAT-R_2_*, there was a significant difference observed in the *Cold MRI* (P = 0.008) but not in the *Reheated MRI*. When comparing sBAT volumes, the sBAT VOIs obtained from image registration were smaller (approximately 4%) than those obtained from manual outlining, both in the *Cold MRI* (P = 0.008) and the *Reheated MRI* (P = 0.011).

## Discussion

The present study estimated, after three hours of mild cold exposure, a mean decrease of approximately -2 pp (P = 0.021) in fat fraction (FF) of cervical-supraclavicular adipose tissue (sBAT-FF), probably caused by active BAT contained within the tissue. No corresponding change in SAT-FF (P = 0.314) was observed. The sustained effect after subsequent reheating indicated the change in sBAT-FF as being mainly caused by lipid consumption rather than by perfusion. In other words, the combustion of lipid stores to produce heat in active BAT was a more likely cause to the decreased fat content (relative to water content) than was increased perfusion associated with BAT activity. To the best of our knowledge, this MRI study is the first to quantify the change in cervical-supraclavicular adipose tissue FF after cold exposure.

The tendency of increasing sBAT-R_2_* and the increase in SAT-R_2_*, observed after cold exposure, could be discussed in terms of e.g. altered blood oxygenation, perfusion and lipid content. However, the true origin of changes in R_2_* during cold exposure is yet not fully understood [12] and warrants further studies e.g. with simultaneous MR or PET perfusion measurements.

The introduction of reheating, for estimation of the contribution from perfusion, resulted in a group average sBAT-FF that remained decreased after reheating (P = 0.008 compared to the *Baseline MRI* and P = 0.953 compared to the *Cold MRI*). This result indicated lipid consumption as the primary cause to the decreased sBAT-FF. However, separation between the two contributions was based on the assumption that the lipid content was more slowly regulated than the perfusion. As this assumption is not established and as perfusion was not measured (using a reference method), in-depth conclusions from reheating could not be drawn. After reheating, mean SAT-FF was observed to decrease in all subjects, by an average of approximately -0.8 pp (P = 0.008). This decrease was thought to be an effect of experimental procedures not related to ambient temperature. These procedures involved a supine position of the subjects throughout the *Cold MRI*, the 15 min of reheating and the following *Reheated MRI* (total time between scans was approximately 30 min). A possible explanation is that the supine position could have caused redistribution of water in the body, compared to a sitting or standing position, which resulted in a decreased SAT-FF. This explanation was supported by results from the *Procedure study* where a similar decrease in SAT-FF (approximately -0.8 pp, P = 0.012) was observed despite unchanged ambient temperature. As no corresponding effect was observed in neither sBAT-FF, sBAT-R_2_* nor SAT-R_2_* (Table 2), the supine position was probably not affecting the sBAT measurements. Hence, the results from the *Procedure study* demonstrate a measurable decrease in posterior SAT-FF when a subject is lying on the back during approximately 30 min but no corresponding effect on sBAT-FF or sBAT-R_2_*.

Regarding R_2_*, a weak trend of sBAT-R_2_* normalization was observed after reheating (P = 0.441 compared to the *Baseline MRI* and P = 0.173 compared to the *Cold MRI*) whereas SAT-R_2_* remained increased (P = 0.028 compared to the *Baseline MRI* and P = 0.767 compared to the *Cold MRI*). How these changes in R_2_* are related to altered blood oxygenation, perfusion and lipid content during BAT activation needs further evaluation e.g. with perfusion measurements.

The present study demonstrates the potential of the *Cooling-reheating protocol* for studies of BAT in humans. Although reference knowledge (from e.g. biopsy, PET/CT) was lacking, the prevalence of cold-activated BAT was expected to be relatively high in the present group of subjects. A prevalence of cold-activated supraclavicular BAT of approximately 50% (in the twenties) and 40% (in the thirties), after a two-hour long mild cold exposure, is reported from a previous PET/CT study [1].

Comparison of sBAT and SAT at baseline, for the estimation of BAT amount, resulted in a trend of lower sBAT-FF compared to SAT-FF (P = 0.139) and a weak trend of higher sBAT-R_2_* than SAT-R_2_* (P = 0.173). These trends were expected, with reference to the microscopic structure of BAT and SAT, and also in line with previously reported results from studies in children and adolescents [10, 11]. However, in the present study a difference in FF and R_2_* between sBAT and SAT could not be statistically established. One likely reason is that the individual BAT amount and location was not known, causing an underestimation of the difference between BAT and SAT and further a group difference below detection limit. Another reason could be that FF and R_2_* are not BAT-specific biomarkers but only reflect the expected lower fat content and higher iron content in BAT than WAT (SAT). When inactive, the morphology of brown adipocytes becomes similar to that of white adipocytes [26]. If this conversion results in a no longer observable difference in FF and R_2_* between BAT and WAT, the tissues become indistinguishable from each other by the method presented. Other physiological properties or conditions, e.g. inflammation or edema, could also alter FF and/or R_2_* and change the relationship between the two tissue types. In addition, very small amounts of BAT intermixed with WAT could remain undetected due to partial volume effects. The reason for the deviating pattern observed in Subj 4 and 7, as compared to the rest of the subjects, is unknown and warrants further evaluation.

In validating the registration method, results obtained from registered data agreed well with results obtained from manually outlined data. However, a difference in sBAT-R_2_* of unknown source was observed in the *Cold MRI* data (P = 0.008). In addition, sBAT VOIs obtained from image registration were smaller than those obtained from manual outlining (approximately 4%, P = 0.008 in the *Cold MRI* and P = 0.011 in the *Reheated MRI*). This was possibly due to differences in subject positioning and scan positioning between the *Baseline*, *Cold* and *Reheated MRI*, causing mismatches of single slices between the scans. These mismatches likely led to partial loss of the manual sBAT VOIs when coregistered with the *Cold* and *Reheated MRI* data, resulting in a systematic difference in the overlap of the sBAT VOIs. However, the conclusions drawn from registered data were consistent with those drawn from manually outlined data.

As the cervical-supraclavicular depot likely contains a mixture of brown adipocytes and white adipocytes [17], partial volume effects are expected to affect the sBAT measurements. Border voxels to large vessels and surrounding tissues are additional sources of partial volume effects in both sBAT and SAT. By prioritizing high-resolution imaging and by adding erosion to the segmented VOIs, the effects were reduced in the present study but not completely removed. A higher resolution is expected to result in exclusion of a larger fraction of the vessels within sBAT and SAT, resulting in a higher FF in both tissue types. As BAT is more densely vascularized than SAT, the reduction of partial volume effects would probably have a greater impact on sBAT than SAT, leading to a smaller difference in FF between the two tissue types. This might have been the reason for the relatively high and similar values of sBAT-FF and SAT-FF as compared to a previous study in young adults (18–30 years) where larger voxels were used [12]. The prevalence of active BAT is observed to decrease with age, at least in adult humans [1]. Therefore, the relatively high mean age of the subjects is a possible explanation for the non-significant difference between sBAT-FF and SAT-FF observed in the present study as compared to a previous study in children and adolescents (9–19 years) [11]. The R_2_* estimates obtained in the present study (performed at 1.5 T) are difficult to compare with those obtained in earlier studies (performed at 3.0 T) due to dependence of R_2_* on magnetic field strength (flux density).

There were some limitations to the present work. The study was lacking a control group, undergoing all protocol procedures besides the cold exposure. Due to absence of such controls, the changes observed in FF and R_2_* after cooling and subsequent reheating could not be established as resulting exclusively from ambient temperature. Other potentially confounding factors, such as length of fasting, could also have influenced the measurements. Further limitations of the present study were those of motion artifacts and of only partial cervical-supraclavicular depot coverage (Fig 2A), being a result of time constraints. Despite the relatively short acquisition time (less than 5 min) and the breathing instructions provided to the subjects, minor artifacts were visually observed in the R_2_* maps of three subjects.

Future work in validating the *Cooling-reheating protocol* is warranted. Validation could preferably be accomplished with a PET/MR system allowing simultaneous data acquisition for the estimation of FF, R_2_*, perfusion and BAT activity.

Data to support the assumptions of perfusion, as being rapidly regulated, and lipid content, as being slowly regulated, are of particular interest. Such data could be acquired by performing water-fat MRI measurements simultaneously with perfusion measurements: at baseline and at multiple time points after both cold exposure and reheating, for studying the temporal pattern of perfusion and FF.

In conclusion, the present study quantified an approximate 2 pp reduction in cervical-supraclavicular (suspected BAT, sBAT) FF during a three-hour long mild cold exposure, accompanied by a trend of increasing R_2_*. The present study also demonstrated the use of a three-step *Cooling-reheating protocol* and presented preliminary results indicating cold-induced BAT activity and the resultant reduction in sBAT-FF as being mainly due to lipid consumption rather than to perfusion.

## Supporting Information

S1 Table Basic characteristics of the subjects. (DOCX)

S2 Table *Cooling-reheating protocol* data from registered and automatically segmented volumes of interest (VOIs). Data from registered cervical-supraclavicular adipose tissue (considered as suspected brown adipose tissue, denoted sBAT) VOI measurements and automatically segmented subcutaneous adipose tissue (SAT) VOI measurements in fat fraction (FF) and R_2_* maps.
(DOCX)

S3 Table *Procedure study* data from registered and automatically segmented volumes of interest (VOIs). Data from registered cervical-supraclavicular adipose tissue (considered as suspected brown adipose tissue, denoted sBAT) VOI measurements and automatically segmented subcutaneous adipose tissue (SAT) VOI measurements in fat fraction (FF) and R_2_* maps.
(DOCX)

S4 Table *Cooling-reheating protocol* data from manually outlined volumes of interest (VOIs). Data from manually outlined cervical-supraclavicular adipose tissue (considered as suspected brown adipose tissue, denoted sBAT) VOI measurements in fat fraction (FF) and R_2_* maps.
(DOCX)

S1 Text Supplementary information to Materials and Methods—Image analysis. (DOCX)
