# Supplementary material for: Magnetic Resonance Imaging Cooling-Reheating Protocol Indicates Decreased Fat Fraction via Lipid Consumption in Suspected Brown Adipose Tissue
Source: PLoS One. 2015 Apr 30;10(4):e0126705. doi: 10.1371/journal.pone.0126705 (PMC4415932; doi:10.1371/journal.pone.0126705)
Supplement: S1 Table — (DOCX) [file pone.0126705.s001.docx]

Supplementary Table 1: Basic characteristics of the subjects.

| Subject | Gender (F/M) | Age [y] | Weight [kg] | Height [m] | BMI [kg/m^2^] |
| --- | --- | --- | --- | --- | --- |
| Subj1 | F | 37 | 61.1 | 1.690 | 21.4 |
| Subj2 | M | 25 | 81.5 | 1.820 | 24.6 |
| Subj3 | F | 22 | 80.6 | 1.795 | 25.0 |
| Subj4 | M | 34 | 75.6 | 1.730 | 25.3 |
| Subj5 | M | 26 | 95.4 | 1.925 | 25.7 |
| Subj6 | M | 35 | 83.1 | 1.855 | 24.1 |
| Subj7 | M | 33 | 70.9 | 1.845 | 20.8 |
| Subj8 | F | 29 | 59.0 | 1.575 | 23.8 |
| Subj9 | F | 26 | 55.9 | 1.755 | 18.1 |
