# Supplementary material for: Magnetic Resonance Imaging Cooling-Reheating Protocol Indicates Decreased Fat Fraction via Lipid Consumption in Suspected Brown Adipose Tissue
Source: PLoS One. 2015 Apr 30;10(4):e0126705. doi: 10.1371/journal.pone.0126705 (PMC4415932; doi:10.1371/journal.pone.0126705)
Supplement: S2 Table — Data from registered cervical-supraclavicular adipose tissue (considered as suspected brown adipose tissue, denoted sBAT) VOI measurements and automatically segmented subcutaneous adipose tissue (SAT) VOI measurements in fat fraction (FF) and R2* maps. (DOCX) [file pone.0126705.s002.docx]

Supplementary Table 2: *Cooling-reheating protocol* data from registered and automatically segmented volumes of interest (VOIs).

| Subject | Scan | sBAT-FF [%] | sBAT-R_2_* [s^-1^] | sBAT-volume [cm^3^] | SAT-FF [%] | SAT-R_2_* [s^-1^] | SAT-volume [cm^3^] |
| --- | --- | --- | --- | --- | --- | --- | --- |
|  |  |  |  |  |  |  |  |
| Subj1 | Baseline | 80.12 | 21.16 | 29.40 | 84.59 | 17.98 | 47.91 |
| Subj1 | Cold | 80.52 | 20.93 | 29.80 | 85.38 | 17.90 | 47.87 |
| Subj1 | Reheated | 79.23 | 20.93 | 28.27 | 84.81 | 17.56 | 49.79 |
|  |  |  |  |  |  |  |  |
| Subj2 | Baseline | 88.66 | 20.78 | 116.73 | 91.81 | 16.79 | 143.77 |
| Subj2 | Cold | 85.20 | 23.40 | 105.29 | 92.16 | 17.07 | 135.28 |
| Subj2 | Reheated | 86.73 | 19.77 | 113.31 | 91.02 | 16.88 | 136.88 |
|  |  |  |  |  |  |  |  |
| Subj3 | Baseline | 84.53 | 19.98 | 45.91 | 89.79 | 17.79 | 74.24 |
| Subj3 | Cold | 82.64 | 20.07 | 45.85 | 89.42 | 18.98 | 79.52 |
| Subj3 | Reheated | 83.05 | 19.35 | 45.93 | 88.33 | 18.77 | 82.31 |
|  |  |  |  |  |  |  |  |
| Subj4 | Baseline | 82.22 | 21.68 | 27.62 | 82.15 | 23.62 | 26.82 |
| Subj4 | Cold | 80.85 | 21.43 | 27.85 | 82.51 | 23.70 | 23.60 |
| Subj4 | Reheated | 80.51 | 22.78 | 26.53 | 81.45 | 24.96 | 19.53 |
|  |  |  |  |  |  |  |  |
| Subj5 | Baseline | 87.03 | 19.45 | 38.41 | 90.16 | 19.23 | 111.84 |
| Subj5 | Cold | 86.12 | 20.26 | 38.66 | 89.89 | 19.58 | 125.62 |
| Subj5 | Reheated | 86.93 | 20.19 | 38.21 | 89.21 | 19.66 | 129.94 |
|  |  |  |  |  |  |  |  |
| Subj6 | Baseline | 82.32 | 20.94 | 34.83 | 84.41 | 20.95 | 47.61 |
| Subj6 | Cold | 80.90 | 21.49 | 36.09 | 84.45 | 20.84 | 48.34 |
| Subj6 | Reheated | 82.01 | 20.75 | 34.46 | 83.60 | 21.03 | 48.20 |
|  |  |  |  |  |  |  |  |
| Subj7 | Baseline | 87.53 | 19.10 | 21.36 | 79.60 | 22.99 | 13.55 |
| Subj7 | Cold | 87.57 | 19.35 | 19.31 | 80.87 | 23.21 | 15.45 |
| Subj7 | Reheated | 86.65 | 19.51 | 18.46 | 80.56 | 23.17 | 13.44 |
|  |  |  |  |  |  |  |  |
| Subj8 | Baseline | 80.68 | 20.66 | 33.14 | 87.67 | 15.69 | 79.81 |
| Subj8 | Cold | 77.00 | 21.57 | 30.53 | 87.42 | 16.39 | 74.61 |
| Subj8 | Reheated | 76.79 | 21.18 | 29.53 | 86.75 | 16.59 | 78.98 |
|  |  |  |  |  |  |  |  |
| Subj9 | Baseline | 72.40 | 25.20 | 19.94 | 76.98 | 21.77 | 6.18 |
| Subj9 | Cold | 67.25 | 26.30 | 13.44 | 77.16 | 22.71 | 5.03 |
| Subj9 | Reheated | 66.33 | 25.85 | 13.33 | 76.38 | 22.41 | 6.56 |
