# Supplementary material for: Magnetic Resonance Imaging Cooling-Reheating Protocol Indicates Decreased Fat Fraction via Lipid Consumption in Suspected Brown Adipose Tissue
Source: PLoS One. 2015 Apr 30;10(4):e0126705. doi: 10.1371/journal.pone.0126705 (PMC4415932; doi:10.1371/journal.pone.0126705)
Supplement: S3 Table — Data from registered cervical-supraclavicular adipose tissue (considered as suspected brown adipose tissue, denoted sBAT) VOI measurements and automatically segmented subcutaneous adipose tissue (SAT) VOI measurements in fat fraction (FF) and R2* maps. (DOCX) [file pone.0126705.s003.docx]

Supplementary Table 3: *Procedure study* data from registered and automatically segmented volumes of interest (VOIs).

| Subject | Scan | sBAT-FF [%] | sBAT-R_2_* [s^-1^] | sBAT-volume [cm^3^] | SAT-FF [%] | SAT-R_2_* [s^-1^] | SAT-volume [cm^3^] |
| --- | --- | --- | --- | --- | --- | --- | --- |
|  |  |  |  |  |  |  |  |
| Subj1 | Scan1 | 78.68 | 21.43 | 28.47 | 86.62 | 17.02 | 63.85 |
| Subj1 | Scan2 | 79.33 | 20.24 | 28.54 | 86.04 | 16.93 | 64.83 |
|  |  |  |  |  |  |  |  |
| Subj2 | Scan1 | 86.88 | 21.48 | 114.90 | 92.29 | 16.98 | 160.63 |
| Subj2 | Scan2 | 86.75 | 22.77 | 109.17 | 92.19 | 17.07 | 165.64 |
|  |  |  |  |  |  |  |  |
| Subj3 | Scan1 | 82.98 | 19.32 | 39.65 | 88.83 | 18.06 | 65.13 |
| Subj3 | Scan2 | 82.76 | 19.47 | 39.12 | 88.22 | 17.89 | 66.89 |
|  |  |  |  |  |  |  |  |
| Subj4 | Scan1 | 80.67 | 21.09 | 29.40 | 82.54 | 23.24 | 30.51 |
| Subj4 | Scan2 | 80.20 | 21.84 | 28.69 | 80.75 | 24.03 | 27.58 |
|  |  |  |  |  |  |  |  |
| Subj5 | Scan1 | 86.17 | 21.94 | 35.92 | 89.46 | 19.47 | 115.54 |
| Subj5 | Scan2 | 87.35 | 23.42 | 33.71 | 89.19 | 19.63 | 119.22 |
|  |  |  |  |  |  |  |  |
| Subj6 | Scan1 | 81.49 | 20.84 | 31.13 | 84.08 | 21.18 | 54.51 |
| Subj6 | Scan2 | 82.16 | 21.10 | 29.68 | 83.69 | 20.76 | 53.99 |
|  |  |  |  |  |  |  |  |
| Subj7 | — | — | — | — | — | — | — |
| Subj7 | — | — | — | — | — | — | — |
|  |  |  |  |  |  |  |  |
| Subj8 | Scan1 | 78.00 | 21.94 | 30.19 | 87.20 | 16.81 | 89.83 |
| Subj8 | Scan2 | 77.64 | 21.80 | 30.28 | 86.62 | 16.88 | 92.52 |
|  |  |  |  |  |  |  |  |
| Subj9 | Scan1 | 72.24 | 23.98 | 19.60 | 76.76 | 22.41 | 9.22 |
| Subj9 | Scan2 | 70.72 | 23.38 | 19.47 | 75.05 | 22.11 | 9.66 |

Note: “—“ indicates that the subject has not been imaged.
