# Supplementary material for: Magnetic Resonance Imaging Cooling-Reheating Protocol Indicates Decreased Fat Fraction via Lipid Consumption in Suspected Brown Adipose Tissue
Source: PLoS One. 2015 Apr 30;10(4):e0126705. doi: 10.1371/journal.pone.0126705 (PMC4415932; doi:10.1371/journal.pone.0126705)
Supplement: S4 Table — Data from manually outlined cervical-supraclavicular adipose tissue (considered as suspected brown adipose tissue, denoted sBAT) VOI measurements in fat fraction (FF) and R2* maps. (DOCX) [file pone.0126705.s004.docx]

Supplementary Table 4: *Cooling-reheating protocol* data from manually outlined volumes of interest (VOIs).

| Subject | Scan | sBAT-FF [%] | sBAT-R_2_* [s^-1^] | sBAT-volume [cm^3^] |
| --- | --- | --- | --- | --- |
|  |  |  |  |  |
| Subj 1 | Baseline | 80.12 | 21.16 | 29.40 |
| Subj 1 | Cold | 80.46 | 20.81 | 31.54 |
| Subj 1 | Reheated | 79.12 | 20.85 | 30.49 |
|  |  |  |  |  |
| Subj 2 | Baseline | 88.66 | 20.78 | 116.73 |
| Subj 2 | Cold | 85.19 | 23.40 | 109.91 |
| Subj 2 | Reheated | 86.72 | 19.80 | 118.04 |
|  |  |  |  |  |
| Subj 3 | Baseline | 84.53 | 19.98 | 45.91 |
| Subj 3 | Cold | 82.65 | 20.05 | 46.05 |
| Subj 3 | Reheated | 83.07 | 19.29 | 46.70 |
|  |  |  |  |  |
| Subj 4 | Baseline | 82.22 | 21.68 | 27.62 |
| Subj 4 | Cold | 80.95 | 21.42 | 28.70 |
| Subj 4 | Reheated | 80.51 | 22.82 | 27.61 |
|  |  |  |  |  |
| Subj 5 | Baseline | 87.03 | 19.45 | 38.41 |
| Subj 5 | Cold | 86.13 | 20.24 | 40.35 |
| Subj 5 | Reheated | 86.90 | 20.10 | 40.22 |
|  |  |  |  |  |
| Subj 6 | Baseline | 82.32 | 20.94 | 34.83 |
| Subj 6 | Cold | 80.88 | 21.45 | 38.19 |
| Subj 6 | Reheated | 81.94 | 20.76 | 35.87 |
|  |  |  |  |  |
| Subj 7 | Baseline | 87.53 | 19.10 | 21.36 |
| Subj 7 | Cold | 87.47 | 19.21 | 19.76 |
| Subj 7 | Reheated | 86.54 | 19.29 | 17.90 |
|  |  |  |  |  |
| Subj 8 | Baseline | 80.68 | 20.66 | 33.14 |
| Subj 8 | Cold | 77.13 | 21.47 | 31.66 |
| Subj 8 | Reheated | 76.91 | 21.17 | 30.88 |
|  |  |  |  |  |
| Subj 9 | Baseline | 72.40 | 25.20 | 19.94 |
| Subj 9 | Cold | 67.25 | 26.05 | 13.94 |
| Subj 9 | Reheated | 66.10 | 25.74 | 14.45 |
