# Supplementary material for: Magnetic Resonance Imaging Cooling-Reheating Protocol Indicates Decreased Fat Fraction via Lipid Consumption in Suspected Brown Adipose Tissue
Source: PLoS One. 2015 Apr 30;10(4):e0126705. doi: 10.1371/journal.pone.0126705 (PMC4415932; doi:10.1371/journal.pone.0126705)
Supplement: S1 Text — (DOCX) [file pone.0126705.s005.docx]

**Supplementary information**

**Materials and Methods ⎯ Image analysis**

Volumes of interest (VOIs) of suspected brown adipose tissue (hereafter denoted sBAT VOIs) were defined semi-automatically in cervical-supraclavicular adipose tissue in the *Baseline MRI* data sets. Transfer of the sBAT VOIs to remaining data sets was accomplished by image registration. Volumes of white adipose tissue (WAT) were defined fully automatically in posterior subcutaneous adipose tissue (SAT VOIs) without using spatial registration. In the registration and tissue segmentation, computed body and background objects (C_BODY_ and C_BG_, respectively) were used. These were created for all data sets by clustering the sum of the water and fat images.

The sBAT VOIs were constructed from anatomically defined regions of interest, manually outlined in axial slices using ImageJ [1], on which strict exclusion criteria subsequently were applied. The manual delineation resulted in crude sBAT VOIs covering the bilateral cervical-supraclavicular depot. The delineation was performed in the *Baseline MRI* fat fraction (FF) maps meanwhile also considering the corresponding water images and R_2_* maps. The crude sBAT VOIs were defined to include adipose tissue located in between the clavicula and scapula, and to exclude subcutaneous adipose tissue, bone marrow, paravertebral fat and obvious intramuscular fat.

In image registration, a target (moving) volume was deformed to match a reference (fixed) volume by optimization of a cost function. A two-step image registration method was performed using the ITK-based Elastix software [2]. In the first step, the normalized correlation between the C_BODY_s was optimized under an affine deformation. In the second step, a weighted sum of the normalized correlation between the C_BODY_s and the FF maps was optimized under a deformable deformation. For each subject, the resulting deformation was used to transfer the sBAT VOI from the *Baseline MRI* data to the *Cold and Reheated MRI* data (and the *Procedure study* data) of the same subject. Subsequently, segmentation of the adipose tissue within the sBAT VOIs was performed automatically for each data set. This was accomplished by range limits on FF (≥ 40%), followed by morphological 3D erosion (with a six-neighborhood structuring element) and range limits on R_2_* (≤50 s^-1^) for reduction of partial volume effects and boundary voxels between adjacent tissues.

The SAT VOIs were defined posteriorly in all MRI data sets, using an automatic segmentation algorithm. The segmentation was obtained by defining the superficial boundary (towards the skin) and deep boundary (towards the inner tissues) of SAT, followed by the same criteria regarding erosion and range limits on FF and R_2_* as was applied to the sBAT VOIs. The superficial boundary of each SAT VOI was determined from the contour of the C_BODY_, after removal of skin through erosion. The deep boundary of each SAT VOI was determined by identifying the contour of the largest connected body object within the superficial boundary, after removal of fatty tissue. In detail, this contour was obtained according to the following steps: 1) An inverse adipose tissue mask (IATM) (adipose tissue = 0, remaining structures = 1) was created from the C_BG_ by adding lean tissue (defined by FF≤40%) and boundary voxels between adjacent tissues (defined by R_2_*≥50 s^-1^). 2) Background and skin were removed from the IATM by omitting voxels outside the superficial boundary (as defined previously). 3) The deep boundary was defined as the posterior border of the largest connected object that could be retrieved within the IATM, thereby disregarding residual voxels containing vessels, fasciae (located within SAT) and skin. The voxels located posteriorly and in between the superficial and deep boundary were considered as constituting the SAT VOI. By a subsequent morphological opening operation (erosion followed by dilation), unwanted leaks of the VOIs into deeper tissue structures other than SAT could be diminished.

**References**

1. Schneider CA, Rasband WS, Eliceiri KW (2012) NIH Image to ImageJ: 25 years of image analysis. Nat Methods 9: 671-675.
2. Klein S, Staring M, Murphy K, Viergever MA, Pluim JP (2010) elastix: a toolbox for intensity-based medical image registration. IEEE Trans Med Imaging 29: 196-205.
